# The AST/ALT (De-Ritis) ratio: A novel marker for critical limb ischemia in peripheral arterial occlusive disease patients

**DOI:** 10.1097/MD.0000000000003843

**Published:** 2016-06-17

**Authors:** Peter Rief, Martin Pichler, Reinhard Raggam, Franz Hafner, Armin Gerger, Philipp Eller, Marianne Brodmann, Thomas Gary

**Affiliations:** a Division of Vascular Medicine; b Division of Oncology; c Intensive Care Unit, Department of Internal Medicine, Medical University Graz, Graz, Austria.

**Keywords:** AST/ALT ratio, critical limb ischemia, nonalcoholic fatty liver disease, peripheral arterial occlusive disease

## Abstract

The aspartat aminotransferase (AST)/alanin aminotransferase (ALT) (De-Ritis) ratio (AAR) is an easily applicable blood test. An elevated AAR on the one hand has been associated with an increase in nonalcoholic fatty liver disease (NAFLD). NAFLD on the other hand is associated with an increase in cardiovascular disease, all-cause mortality, and diabetes. As the AAR is also elevated in case of muscular damage, we investigated AAR and its association with critical limb ischemia (CLI) in peripheral arterial occlusive disease (PAOD) patients.

In our cross-sectional study, we included 1782 PAOD patients treated at our institution from 2005 to 2010. Patients with chronic alcohol consumption (>20 g/day) were excluded. AAR was calculated and the cohort was categorized into tertiles according to the AAR. An optimal cut-off value for the continuous AAR was calculated by applying a receiver operating curve analysis to discriminate between CLI and non-CLI.

In our cohort, occurrence of CLI significantly increased with an elevation in AAR. As an optimal cut-off value, an AAR of 1.67 (sensitivity 34.1%, specificity 81.0%) was identified. Two groups were categorized, 1st group containing 1385 patients (AAR < 1.67) and a 2nd group with 397 patients (AAR > 1.67). CLI was more frequent in AAR > 1.67 patients (166 [41.9%]) compared to AAR < 1.67 patients (329 [23.8%]) (*P* < 0.001), as was prior myocardial infarction (28 [7.1%] vs 54 [3.9%], *P* = 0.01). Regarding inflammatory parameters, C-reactive protein (median 8.1 mg/L [2.9–28.23] vs median 4.3 mg/L [2.0–11.5]) and fibrinogen (median 427.5 mg/dL [344.25–530.0] vs 388.0 mg/dL [327.0–493.0]) also significantly differed in the 2 patient groups (both *P* < 0.001). Finally, an AAR > 1.67 was associated with an odds ratio (OR) of 2.0 (95% confidence interval [CI] 1.7–2.3) for CLI even after adjustment for other well-established vascular risk factors.

An increased AAR is significantly associated with patients at high risk for CLI and other cardiovascular endpoints. The AAR is a broadly available and cheap marker, which might be useful to highlight patients at high risk for vascular endpoints.

## 1 Introduction

Peripheral arterial occlusive disease (PAOD) is frequent and often not diagnosed in time.^[1]^ If PAOD is not diagnosed and treatment is not initiated immediately, the probability of disease progression and development of critical limb ischemia (CLI) is high.^[2]^ CLI is an entity with high mortality and high risk of limb amputation. Although treatment options, especially endovascular treatment possibilities, improved in the last decades, mortality, and amputation rates are still high.^[3,4]^

Nonalcoholic fatty liver disease (NAFLD) is characterized by excessive accumulation of triglycerides in the liver in absence of excessive alcohol consumption.^[5]^ NAFLD is closely associated with cardiovascular disease and even all-cause mortality.^[6,7]^ The gold standard for the diagnosis of NAFLD is liver biopsy.^[8]^ Because of its limitations and invasiveness it is not performed in clinical practice and surrogate markers, mainly liver enzymes like the aspartat aminotransferase (AST)/alanin aminotransferase (ALT) (De-Ritis) ratio (AAR) are used for the diagnosis of this common disease.^[5]^

NAFLD was already evaluated as a risk factor for vascular endpoints in atherosclerosis patients in various studies. Only recently Fracanzani et al^[9]^ were able to show that NALFD, especially in combination with elevated liver enzymes, was significantly associated with cardiovascular events, defined as acute coronary syndrome, and stroke in a 10 years follow-up. However, similar to other studies performed in the field of atherosclerosis, PAOD patients were not outlined in this study and CLI was not included as an end point as well.

As PAOD is frequent and CLI is an entity with a high mortality, highlighting PAOD patients with a high risk for CLI is of importance. As the AAR is an easy to perform test and AAR is also elevated in case of muscular damage we investigated AAR and its association with CLI in PAOD patients.

## 2 Methods

We included 1782 consecutive PAOD patients treated at our department from 2005 to 2010 in our cross-sectional study. Inclusion criterion for our analysis was treatment at our institution for PAOD during the time period described above. Exclusion criterion in our study was alcohol consumption of >20 g/day, which goes along with international recommendations.^[10]^ The study was approved by the International Review Board of the Medical University of Graz, Austria (IRB Number 24-506 ex 11/12). As this was a retrospective data analysis of blinded data no written or verbal consent was obtained, which was approved by the ethics committee.

The diagnosis and graduation of PAOD was assigned in our outpatient clinic by means of clinical evaluation, ankle brachial index, and duplex scan according to the TASC II criteria. Patients were successive patients admitted to our outpatient clinic because of their PAOD and afterwards scheduled for admission at our ward for further treatment of their atherosclerotic disease. Blood was taken in fasting patients at the time of diagnosis of PAOD and laboratory examinations were performed. PAOD was graduated using Fontaine classification, CLI was defined as PAOD patients presenting with ischemic rest pain and/or skin ulceration/gangrene in accordance to current guidelines reflecting patients with Fontaine class 3 and 4.^[11]^ When patients were admitted to the hospital, the medical records of the patients were analyzed by a standardized questionnaire with attention to cardiovascular risk factors and comorbidities. Clinical symptoms were evaluated and physical examination was performed.

### 2.1 Statistical analyses

Clinical characteristics of subjects were analyzed using descriptive statistics. For comparison of groups Chi-square test for categorical values, *t* test for normally distributed continuous variables, and Mann–Whitney *U* test for nonnormally distributed continuous variables were used.

The study population was divided into tertiles according to their continuous AAR. In order to reveal a statistical trend for AAR and CLI a Jonckheere–Terpstra test was performed. The optimal cut-off value for the continuous AAR was calculated by applying a receiver operating curve analysis to test all possible cut-offs that would discriminate between CLI and non-CLI as previously described.^[12]^

We further calculated odds ratios (ORs) with 95% confidence intervals for different CLI-risk factors with a binary logistic regression model. All tests used a *P*-value of 0.05 as a threshold for significance. All statistical analyses were performed using SPSS 22.0.

## 3 Results

**Figure 1:**
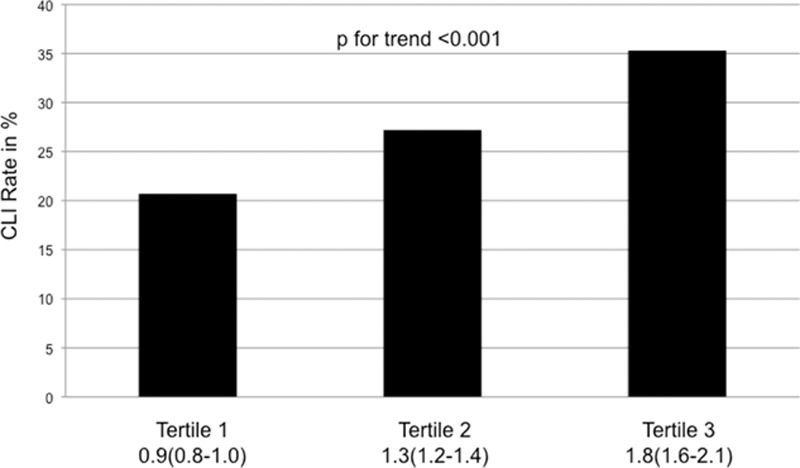
Percentage of patients with critical limb ischemia stratified by tertiles of aspartat aminotransferase/alanin aminotransferase ratio. Numbers below the figure are median aspartat aminotransferase/alanin aminotransferase ratio and the 25th and 75th percentile.

A total of 1782 PAOD patients were included in the current analysis. Patients’ characteristics are shown in Table 1. In a 1st step the study population was categorized according to the AAR into 3 tertiles each containing 594 patients. In the 1st tertile (median AAR 0.9, 0.8–1.0) the CLI rate was 20.7%, in the 2nd tertile (median AAR 1.3, 1.2–1.4) the CLI rate was 27.2%, and in the 3rd tertile (median AAR 1.8, 1.6–2.1) the CLI rate was 35.3% (Fig. 1). In order to evaluate the trend for increase of CLI rate for increasing AAR a Jonckheere–Terpstra test was performed and showed statistical significance (*P* < 0.001).

**Table 1:**
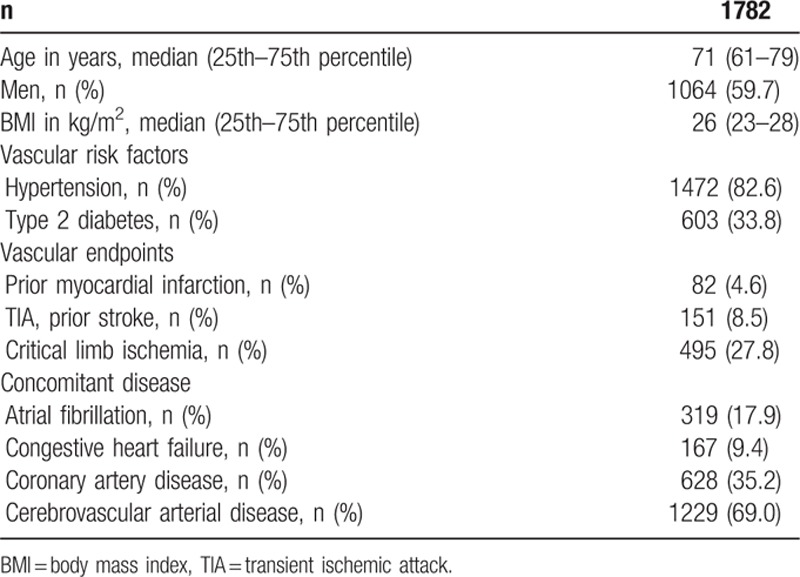
Patients’ characteristics of all peripheral arterial occlusive disease patients included in the study.

In a 2nd step an AAR value of 1.67 was calculated by receiver-operating curve analysis as an optimal cut-off value to discriminate between CLI and non-CLI with a sensitivity of 34.1% and a specificity of 81.0%. Consequently, we categorized our cohort into 2 groups: 1st group with an AAR < 1.67 containing 1385 patients and a 2nd group with an AAR > 1.67 containing 397 patients. The 1st group contained 329 (23.8%) CLI patients whereas the 2nd group included 166 (41.9%) patients with CLI. The difference between groups was statistically significant (*P* < 0.001). Between the 2 AAR groups we found further statistically significant differences in other vascular endpoints (prior myocardial infarction 54 [3.9%] vs 28 [7.1%], *P* = 0.01) and in inflammatory parameters (C-reactive protein [CRP] [median 4.3 mg/L (2.0–11.5) vs 8.1 mg/L (2.9–28.23)] and fibrinogen [median 388.0 mg/dL (327.0–493.0) vs 427.5 mg/dL (344.25–530.0)]; both *P* < 0.001) as well (Table 2). We also did statistical analyses on the correlation of AAR with CRP and fibrinogen. We calculated a Pearson correlation and a Spearman rho as well for AAR and CRP as for AAR and fibrinogen. For both parameters, we could confirm a statistically significant but not relevant correlation between the AAR and CRP/fibrinogen (*P*-value of <0.001; for AAR and CRP: Pearson *r* = 0.18; Spearman rho = 0.14; for AAR and fibrinogen: Pearson *r* = 0.08; Spearman rho = 0.09).

**Table 2:**
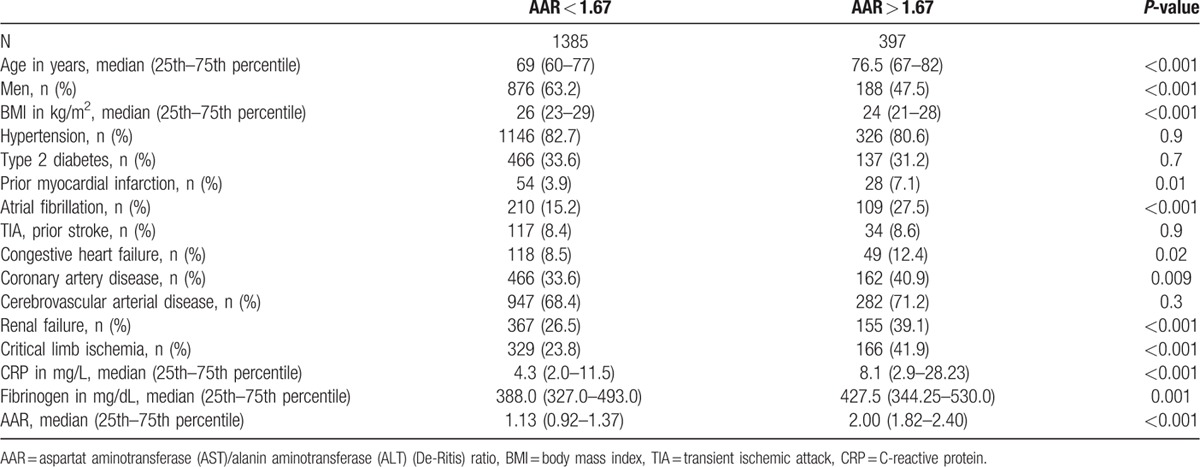
Clinical and hematological characteristics of population with AAR < 1.67 and AAR > 1.67.

In a 3rd step AAR > 1.67 was used as a variable in a binary logistic regression model to evaluate this value as an independent risk factor for CLI. In this model AAR > 1.67, sex, type 2 diabetes, age > 75 years, coexistence of congestive heart failure, arterial hypertension, CRP, and renal impairment were included. Type 2 diabetes, age >75 years, and renal impairment were included as these variables showed a close association with a coexisting CLI in studies published recently from our group^[13,14]^ and CRP was included as this is an established parameter reflecting vascular inflammation.^[15]^ Even after adjustment for these parameters AAR > 1.67 was associated with an OR of 2.0 (95%confidence interval 1.7–2.3, *P* < 0.001) for CLI (Table 3).

**Table 3:**
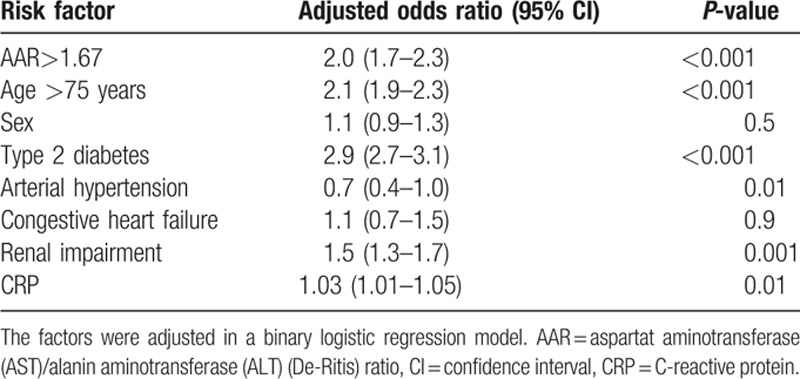
Adjusted risk factors for critical limb ischemia in peripheral arterial occlusive disease patients.

## 4 Discussion

In this study, we were able to demonstrate that an AAR > 1.67 is associated with CLI in PAOD patients. Even after adjustment for other main CLI risk factors like renal impairment, diabetes, and age >75 years, AAR > 1.67 was associated with a 2-fold increase in CLI risk. However, not only CLI was more frequently found in the high AAR group. Endpoints due to atherosclerotic lesions in other vascular beds, like myocardial infarction, were also more frequently encountered in this group. Even entities associated with coronary artery disease, like congestive heart failure and atrial fibrillation,^[16]^ were significantly more prevalent in the group with AAR > 1.67.

NAFLD might be one reason for our findings concerning elevated AAR and association with cardiovascular endpoints in our study. This entity was only recently evaluated in over 3000 patients included in the Framingham Heart Study.^[17]^ The authors were able to show that NAFLD was significantly associated with subclinical cardiovascular outcomes defined as coronary artery calcium and abdominal artery calcium.^[17]^ The mechanisms by which hepatic steatosis might contribute to vascular disease is still under discussion. Of course both entities share various risk factors such as diabetes, elevated triglyzerides, and hypertension. However, Mellinger et al^[17]^ were able to show that the association of NAFLD with subclinical atherosclerosis was independent of these established risk factors. The authors discuss that microRNAs might play a certain role as have been shown to be released by the liver and promote vascular disease.^[18]^

The close correlation of NAFLD with abdominal obesity and insulin resistance makes it difficult to distinguish the causal relationship underlying the increased risk of cardiovascular disease in these patients. Hepatic steatosis is associated with increased production of interleukin-6 and other proinflammatory cytokines by hepatozytes and nonperynchymal cells, including Kupfer cells.^[19–22]^ These increased cytokine expression is likely to play a key role in the progression of NAFLD and cardiovascular disease as well. Several case–control studies were able to show that inflammatory markers also reflecting inflammation in atherosclerosis patients, like CRP, interleukin-6, and fibrinogen were highest in NAFLD patients, intermediate in patients with simple steatosis and lowest in control subjects without steatosis.^[23]^ These differences were independent of obesity and other potentially confounding factors. Increased CRP promotes inflammation and atherosclerosis via increase in expression of plasminogen activator inhibitor-1 and adhesion molecules in endothelial cells and leading to an increase in LDL uptake into macrophages.^[24]^

Of course AAR is influenced by various environmental and physiological parameters. First alcohol intake is a common cause of elevated liver enzymes. As patients with relevant alcohol intake were excluded from our cohort, this influence seems to be negligible in our patients. Second AAR is influenced by body mass index and sex as well. Both serum AST and ALT increase with body weight; however, this seems to be more prominent for ALT rather than AST.^[25]^ Furthermore, the AAR seems to be higher in women.^[26]^ Although both possible confounders were included in our regression analysis, AAR still remained significantly associated with CLI in our cohort.

Interestingly hypertension was associated with an OR of 0.7 with CLI in our regression analysis. We know that hypertension is an established risk factor for atherosclerosis; however, in this analysis it seems to be a protective factor for CLI. Possible explanations for this finding are the prescription of angiotensin-converting enzyme (ACE) or angiotensin II inhibitors, which might lead to a protective effect in PAOD patients.^[27]^ However, an increase in perfusion in the ischemic limb due to arterial hypertension might be protective for the development of CLI as well. In some centers, blood pressure levels in CLI patients are therefore allowed to be above recommended levels in order to increase perfusion of the ischemic limb until ulcer healing has occurred.^[28]^

Our study has several drawbacks: first the retrospective study design. Second we used a single blood sample to calculate AAR. It therefore remains unclear whether this single blood sample reflects an elevated AAR over time. Third, one main reason for the close association of AAR with CLI might be the correlation of AAR with muscle-damage. AST is found (in a significantly higher concentration than ALT) in the muscle and can be released in case of muscular training or muscle damage, as is the case in CLI.^[29–31]^ In this case, AST is more elevated than ALT and therefore the AAR is augmented. However, so far creatine kinase is still the enzyme of choice to evaluate muscular damage, as levels of creatine kinase in muscle are still higher than the levels of the AST and ALT.^[32]^

However, we were able to show that AAR >1.67 can be used to discriminate patients at high risk for CLI from those with a low CLI-risk. Especially in combination with renal function, diabetes, and the age of the patients a discrimination of PAOD patients with high CLI risk seems possible.
